# Diastolic and systolic blood pressure and gout: a Mendelian randomization study

**DOI:** 10.3389/fendo.2024.1367621

**Published:** 2024-05-22

**Authors:** Yanfang Li, Yufeng Xie, Jun Li, Zhichun Chang, Jianmei Zhang, Zunming Zhou, Rong Ren, Yun Chen

**Affiliations:** ^1^Shenzhen Hospital of Guangzhou University of Chinese Medicine (Futian), Shenzhen, China; ^2^The Sixth Clinical Medical College, Guangzhou University of Chinese Medicine, Shenzhen, China; ^3^Faculty of Chinese Medicine and State Key Laboratory of Quality Research in Chinese Medicines, Macau University of Science and Technology, Macao, Macao SAR, China

**Keywords:** diastolic and systolic blood pressure, gout, Mendelian randomization, causality, two-sample

## Abstract

**Background:**

Although there is solid epidemiological evidence supporting the connection between hypertension and gout, little has been said about the relationship between diastolic and systolic blood pressure and gout, the causal relationship and direction associated are uncertain, so we aim to research the causal relationship between diastolic and systolic blood pressure and gout.

**Methods:**

We conducted a two-sample Mendelian randomization (MR) analysis to assess the causal effect between 2 blood pressure phenotypes (including diastolic blood pressure and systolic blood pressure) and 5 gout phenotypes (including gout, drug-induced gout, idiopathic gout, unspecified gout, and strictly defined gout) using genome-wide association study statistics. The inverse variance weighting method was used to generate the main results, while sensitivity analyses using MR-Egger, weighted median, Cochran’s Q test, Egger intercept test, and leave-one-out analysis, were performed to assess the stability and reliability of the results.

**Results:**

After the screening, we found a causal relationship between diastolic blood pressure and gout, idiopathic gout, unspecified gout, and strictly defined gout, and a causal relationship between systolic blood pressure and gout, idiopathic gout, unspecified gout, and strictly defined gout.

**Conclusion:**

From a genetic predisposition, controlling blood pressure may reduce the risk of gout.

## Introduction

Gout is a condition caused by monosodium urate crystals deposition in tissues, which is featured by hyperuricemia resulting from purine metabolism disorders and reduced uric acid excretion (1). It is the most common form of inflammatory arthritis, with about 41 million adults worldwide affected. Recent reports of the prevalence and incidence of gout vary widely according to the population studied and methods employed but range from a prevalence of <1% to 6.8% and an incidence of 0.58–2.89 per 1,000 person-years (2). As reported, the global burden of gout has been increasing over the past three decades, but the strategies for its management remain suboptimal (3). Thereby, there is an urgent need to develop strategies to improve the quality of gout care (4).

Most observational studies have shown that hypertension is strongly associated with the incidence of gout (5–9). For example, a study based on primary care data from the United Kingdom found that people with gout were one time more likely to have hypertension at diagnosis than controls (10). In the Normative Ageing Study, the incidence of gout in hypertensive patients is three times that of non-hypertensive patients (8). In addition, previous studies also reported that people with hypertension are generally twice as likely to develop gout than those without hypertension (11).

Blood pressure consists of systolic and diastolic blood pressure, and while the number of studies on hypertension and gout has increased in recent years, few articles separate systolic and diastolic blood pressure to study the relationship with gout (12). Given the uncertainty of the causal relationship between systolic blood pressure, diastolic blood pressure, and gout, we refer to the potential causal impact of systolic blood pressure and diastolic blood pressure on the development of gout as assessed by Mendelian randomization (MR) analyses using data from large-scale genome-wide association studies (13, 14).

MR analysis is a statistical method based on genome-wide sequencing data, which can effectively reduce bias and can be used to reveal causal relationships, similar to a randomized controlled trial (RCT) design (15, 16). Specifically, MR is a method based on instrumental variables. The instrumental variables must meet the following conditions: (i) the genotype is associated with the exposure; (ii) the genotype is associated with the outcome through the studied exposure only (exclusion restriction assumption); and (iii) the genotype is independent of other factors which affect the outcome (independence assumption) (17). Typically, in an MR design, genetic variants, also called single nucleotide polymorphisms (SNPs), are used as instrumental variables. Based on random assignment at meiosis and fixed assignment to genetic variation at conception, MR studies are less susceptible to confounding factors and reverse causality (18). Therefore, we hope to investigate the causal association between diastolic and systolic blood pressure and the risk of gout using an MR design. This work would further provide evidence for the effect of blood pressure on the risk of gout and would provide novel targets for the prevention of gout (19).

## Methods and materials

### Study design

This study used 2 blood pressure phenotypes and 5 gout phenotypes for Mendelian randomization analysis, the blood pressure phenotypes were obtained from the IEU database (https://gwas.mrcieu.ac.uk/), and the gout phenotypes were obtained from the FinnGen database (20) (FinnGen http://www.finngen.fi/en). Statistical analyses were performed by the “TwoSampleMR” package (version 0.5.6) of the R program (version 4.2.2). Only summary-level statistics were used in our study and therefore ethical approval was not required.

### GWAS data for diastolic and systolic blood pressure

The GWAS summary data for diastolic and systolic blood pressure phenotypes were obtained from the International Consortium of Blood Pressure, Participants used sex, age, age-squared, BMI, and chip as covariates and Meta-analysis of cohort/cross-sectional studies as study design. There were 757,601 European populations of systolic blood pressure Sample size and 7,088,083 SNPs were tested. The diastolic blood pressure phenotypes came from 757,601 European descents and 7160619 SNPs were tested (**Supplementary Material S6**).

### GWAS data for gout, idiopathic gout, unspecified gout, strictly defined gout, and drug-induced gout

Summary statistics for gout, idiopathic gout, unspecified gout, strictly defined gout, and drug-induced gout phenotypes were obtained from the FINNGEN Consortium. They all refer to ICD criteria as diagnostic criteria (**Supplementary Material S6**).

There were 7461 Finnish populations of gout phenotype cases and 221323 controls, 429209 people referred to the ICD criteria for gout as a diagnostic criterion., with a mean age at first event 67.05 years for females and 64.43 years for males.

The Idiopathic gout phenotype, which includes 1851 cases and 335038 controls. 429209 people referred to the ICD criteria for idiopathic gout as a diagnostic criterion, with a mean age at first event (years) of -68.44 years for females and 66.23 years for males.

Drug-induced gout phenotype: There are 110 cases and 342389 controls. 429209 people referred to the ICD criteria for drug-induced gout as a diagnostic criterion, with a mean age at the first event (years) of -70.89 years for females and 67.77 years for males.

Unspecified gout phenotype: There are 4607 cases and 335038 controls. 429209 people referred to the ICD criteria for unspecified gout as a diagnostic criterion, with a mean age at the first event (years) of -69.10 years for females and 67.64 years for males.

Strictly defined gout phenotype: There are 3768 cases and 335038 controls. 429209 people refer to the ICD criteria for unspecified gout as a diagnostic criterion, with a mean age at the first event (years) of -65.70 years for females and 61.58 years for males.

### Mendelian randomization analysis

MR analysis must satisfy the three main assumptions that SNPs are strongly correlated with exposure (correlation assumption), that SNPs are not correlated with outcome, and that SNPs are not correlated with confounders. To satisfy the correlation assumption, we used P < 5 × 10^–8^ and linkage disequilibrium (LD) < 0.001 as SNPs correlated with exposure (diastolic and systolic blood pressure in this study). Through analysis, we obtained 461 SNPs associated with systolic blood pressure strength and 460 SNPs with diastolic blood pressure strength (P < 5 × 10^–8^, LD r^2^ < 0.001). We then extracted the exposure SNPs from the outcome data of five gout phenotypes and excluded those associated with the outcome (P < 5 × 10^-8^). For SNPs absent in the outcome, proxied SNPs were identified in high LD (r^2^ > 0.8) based on the European reference panel of the 1000 Genomes Project. For those absent and no appropriate proxies identified, we discarded them. Harmonization was then conducted to align the alleles of the exposure- and outcome-SNPs, and discard palindromic SNPs with intermediate effect allele frequencies (EAF > 0.42) or SNPs with incompatible alleles (e.g. A/G vs. A/C).

We applied the inverse variance weighted (IVW) estimation in the main analysis, which combines the Wald ratio of each SNP to the outcome to obtain a pooled causal estimate. The IVW method has significantly higher statistical power than other MR methods and is thus typically used as the primary approach (21). Besides, we considered a significant association based on an IVW estimation with P < 0.005 (Bonferroni correction: 0.05/2 exposures/5 outcomes). Two other MR models, including the weighted median (WM) and MR-Egger method, were used as complementary methods. The WM method assumes that at least half of the instruments were valid, and the statistical power is mildly weaker than the IVW method (22). MR-Egger regression provides consistent estimates accounting for pleiotropy when all the instruments are invalid (23).

### Sensitivity analysis

To test the reliability of our MR analysis, a sensitivity analysis was required. We used Cochran’s Q test, leave one out (LOO), and MR-Egger intercept analysis for sensitivity analysis (23). Heterogeneity exists when the P-value of Cochran’s Q test is less than 0.05 (24). We assessed horizontal pleiotropy results based on the intercept term derived from the MR-Egger intercept analysis (23). When P < 0.05 for MR-Egger intercept analysis, there was pleiotropy (25). To determine whether causal estimates were driven by any individual SNPs, we performed a LOO analysis, discarding each exposure-related SNP in turn by LOO, to replicate the IVW analysis.

### Risk factors analysis

Except for a range of statistical methods performed in sensitivity analyses to assess any violations of MR assumptions, we also used the Phenoscanner website (http://www.phenoscanner.medschl.cam.ac.uk/) to see if certain SNPs were associated with other phenotypes serving as potential risk factors of blood pressure and gout, including diabetes, obesity and age. Once SNPs were identified to be associated with potential confounders mentioned above at a threshold of p < 5 × 10^–5^, IVW was repeated after removing these SNPs manually to verify the robustness of the results. To explore the potential mechanisms underlying the phenotypic genetic association of blood pressure and gout, we further conducted MR analyses to investigate the causal effect of blood pressure on several potential mediators, including body mass index (BMI), and smoking. The genetic effects of BMI phenotype came from the Genetics of Anthropometric Traits Research (GIANT) consortium (26). Smoking phenotype GWAS summary data were obtained from the FinnGen database. The IVW method was used as the major analysis, with P < 0.05 considered as a significant association.

## Results

**Figure 1:**
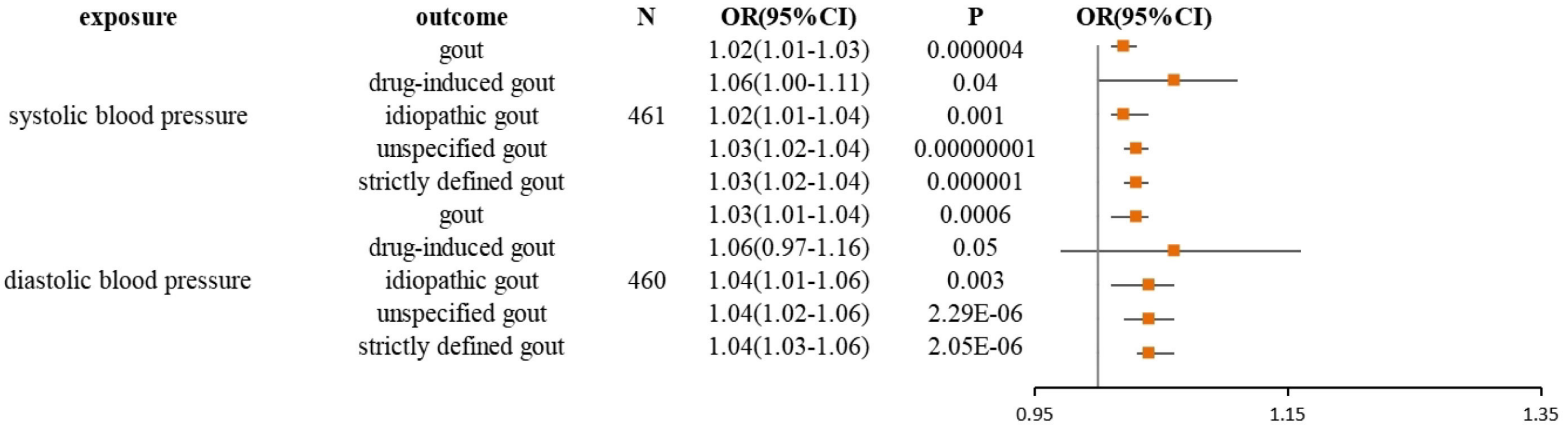
Forest plot for the causal effect of diastolic and systolic blood pressure on the risk of gout, drug-induced gout, idiopathic gout, unspecified gout, and strictly defined gout derived from inverse variance weighted (IVW). OR, odds ratio; CI, confidence interval.

**Figure 2:**
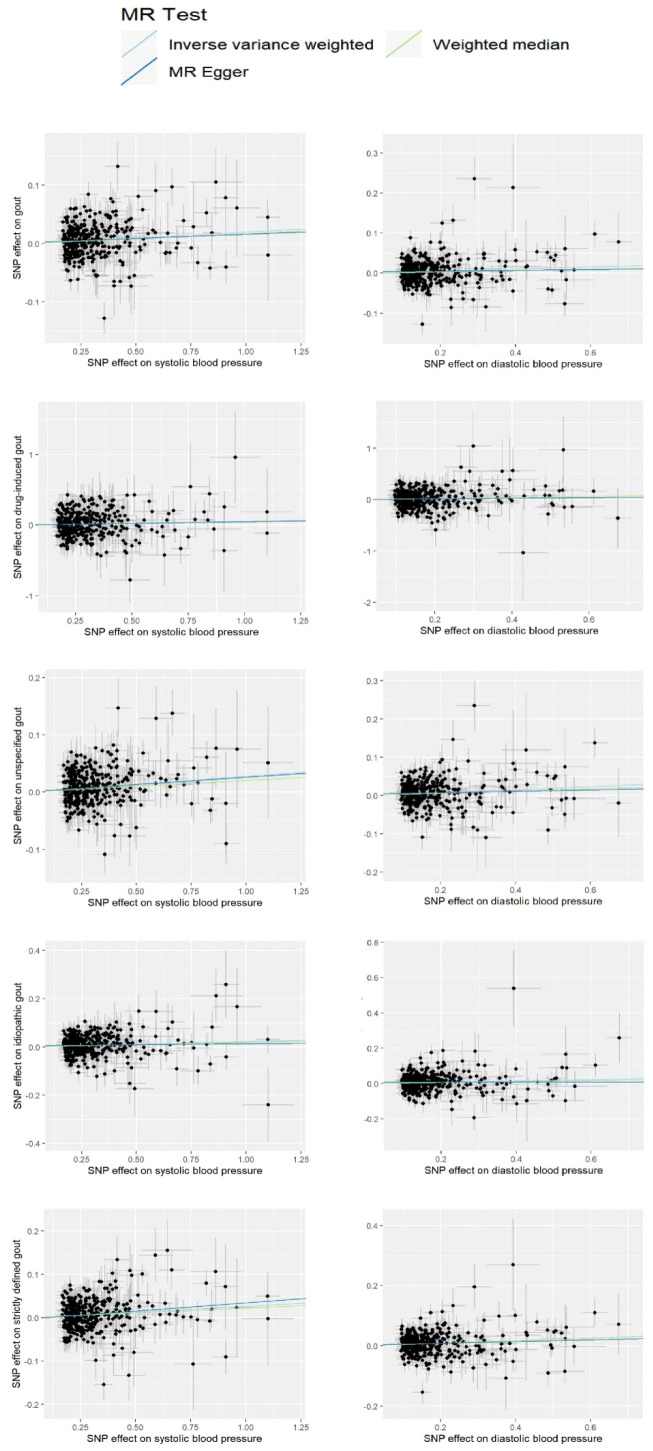
Scatter plot for the significant Mendelian randomization (MR) association between diastolic blood pressure and gout, drug-induced gout, idiopathic gout, unspecified gout, and strictly defined gout; systolic blood pressure and gout, drug-induced gout, idiopathic gout, unspecified gout, and strictly defined gout. SNP, single nucleotide polymorphism.

The current study assessed the causal effect of 2 blood pressure phenotypes on 5 gout phenotypes (**Supplementary Material S1**), and in the preliminary analyses, a total of 9 causal features were identified at P < 0.05 (**Figure 1**). Of the nine initially observed causal features, systolic blood pressure was causally associated with all five gout phenotypes, and diastolic blood pressure was causally associated with four gout phenotypes. After Bonferroni correction, we observed a causal effect of two blood pressure phenotypes on several gout phenotypes in the IVW model. In detail, we found that unspecified gout (OR = 1.03; 95% CI = 1.02 - 1.04; P = 1.00×10^–8^), idiopathic gout (OR = 1.02; 95% CI = 1.01 - 1.04; P = 0.001), gout (OR = 1.02; 95% CI = 1.01 - 1.03; P = 4.00×10^–6^), and strictly defined gout (OR = 1.03; 95% CI = 1.02 - 1.04; P = 1.00×10^–6^) were associated with systolic blood pressure, whereas unspecified gout (OR = 1.02; 95% CI = 1.01 - 1.03; P = 6.66 × 10^–9^), idiopathic gout (OR = 1.04; 95% CI = 1.02 - 1.06; P = 2.29 × 10^–6^), gout (OR = 1.03; 95% CI = 1.01 - 1.04; P = 6.00 ×10^–4^), and strictly defined gout (OR = 1.04; 95% CI = 1.03 - 1.06; P = 2.05 × 10^–6^) was associated with diastolic blood pressure. Heterogeneity (**Supplementary Material S2**) was observed in systolic blood pressure with strictly defined gout(Q = 483.85, P = 0.0003), gout (Q = 575.48, P =1.20 × 10^–11^), unspecified gout (Q = 452.14, P = 0.001), and diastolic blood pressure with strictly defined gout (Q = 496.09, P =2.28 × 10^–5^), gout (Q = 645.25, P= 9.73 × 10^–17^), unspecified gout (Q = 506.37, P = 5.73 × 10^–6^), idiopathic gout (Q = 467.85, P = 0.0007). Although heterogeneity was detected in some of the results, heterogeneity was acceptable as we used random-effect IVW as the main outcome (27). At the same time, we observed that the causal estimates were consistent between the MR models (**Table 1**), and the MR- Egger intercept indicated that no directional pleiotropy was detected (**Figure 2**; **Supplementary Material S3**). In addition, the LOO analyses did not reveal any high-impact SNPs biasing the pooled effect estimates (**Supplementary Material S7**).

**Table 1:** Sensitivity analysis for the causal association between diastolic blood pressure and gout, drug-induced gout, idiopathic gout, unspecified gout, and strictly defined gout; systolic blood pressure and gout, drug-induced gout, idiopathic gout, unspecified gout, and strictly defined gout.

exposure	outcome	WM		MR-egger		Heterogeneity		Pleiotropy	
		OR(95% CI)	p	OR(95% CI)	p	Q	p	Intercept	p
systolic blood pressure	gout	1.02(1.01–1.03)	0.002	1.01(0.99–1.04)	0.21	575.48	1.20E-11	0.0019	0.59
	drug-induced gout	1.04(0.96–1.14)	0.32	1.01(0.99–1.04)	0.37	374.51	0.35	0.0030	0.89
	idiopathic gout	1.02(1.00–1.04)	0.09	1.01(0.97–1.04)	0.65	375.72	0.34	0.0047	0.38
	unspecified gout	1.02(1.01–1.03)	0.002	1.03(1.00–1.05)	0.04	452.14	0.001	0.0006	0.87
	strictly defined gout	1.02(1.01–1.04)	0.005	1.04(1.01–1.07)	0.01	483.85	0.00003	-0.0036	0.40
diastolic blood pressure	gout	1.02(1.00–1.04)	0.1	1.01(0.97–1.05)	0.58	645.25	9.73E-17	0.003	0.35
	drug-induced gout	1.11(0.96–1.28)	0.17	1.07(0.86–1.33)	0.55	377.17	0.4	-0.001	0.96
	idiopathic gout	1.03(0.99–1.06)	0.13	1.00(0.95–1.06)	0.91	467.85	0.0006	0.007	0.23
	unspecified gout	1.03(1.01–1.05)	0.01	1.02(0.98–1.06)	0.38	506.37	5.73E-06	0.004	0.24
	strictly defined gout	1.04(1.02–1.07)	0.002	1.03(0.99–1.07)	0.19	496.09	2.28E-05	0.003	0.48

### Risk factor analysis

By looking over the Phenoscanner, we found some SNPs for systolic and diastolic blood pressure were associated with obesity and diabetes confounding factors. SNP of diastolic blood pressure, (rs2384061、rs2307111、rs962369), were associated with obesity-related traits. SNP of diastolic blood pressure, (rs10804330、rs35261542、rs1265157、rs6905288), were associated with diabetes-related traits. SNP of systolic blood pressure, (rs11672660), was associated with obesity-related traits. SNP of systolic blood pressure, (rs10804330、rs28650790、rs9368222、rs34072724、rs12255372、rs2129869、rs7306710、rs6062324), were associated with diabetes-related traits. Which could potentially bias the overall estimate. We manually eliminate these SNPs and repeated the IVW analysis, and significant or not results are unchanged. unspecified gout (OR = 1.03; 95% CI = 1.02 - 1.04; P = 8.04×10^–^10), idiopathic gout (OR = 1.02; 95% CI = 1.01 - 1.04; P = 0.007), gout (OR = 1.02; 95% CI = 1.01 - 1.03; P = 9.99×10^–^7), and strictly defined gout (OR = 1.03; 95% CI = 1.02 - 1.04; P = 1.82×10^–^7) were still associated with systolic blood pressure. After Bonferroni correction, drug-induced gout is still not significant (OR = 1.06; 95% CI = 1 - 1.12; P = 0.04). whereas unspecified gout (OR = 1.04; 95% CI = 1.03 - 1.06; P = 2.87 × 10^–7^), idiopathic gout (OR = 1.04; 95% CI = 1.02 - 1.07; P = 8.88 × 10^–6^), gout (OR = 1.03; 95% CI = 1.01 - 1.04; P = 6.00 ×10^–4^), and strictly defined gout (OR = 1.05; 95% CI = 1.03 - 1.06; P = 5.13 × 10^–7^) were also associated with diastolic blood pressure. drug-induced gout (OR = 1.07; 95% CI = 0.97 - 1.16; P = 0.17) is still not significant. To further investigate potential mediators linking blood pressure to increased risk of gout, we used MR methods to assess the effects of blood pressure on several common risk factors: systolic blood pressure and smoking (OR = 1.00; 95% CI = 0.99 - 1.02; P = 0.13), systolic blood pressure and BMI (OR = 0.99; 95% CI = 0.991 - 0.998; P = 0.002), diastolic blood pressure and smoking (OR = 1.01; 95% CI:1.00 - 1.03; P = 0.14), and diastolic blood pressure and BMI (OR = 0.98; 95% CI:0.98 - 0.99; P = 3.73 × 10^–5^) (**Supplementary Materials S4**, **S5**).

## Discussion

### Diastolic and systolic blood pressure and gout

Our study showed that a Mendelian analysis with systolic and diastolic blood pressure as exposure and gout as outcome showed a reliable association between them. Elevated systolic and diastolic blood pressure is positively associated with the risk of gout, and previous clinical studies have found a positive correlation between hypertension and hyperuricemia (28). A study found that men with hyperuricemia who had normal blood pressure showed significantly exaggerated systolic or diastolic blood pressure during a maximal treadmill exercise test (29). Previous meta-analyses have also shown that hypertension is associated with elevated serum urate levels (30). Other studies have emphasized that the prognostic significance of hyperuricemia can actually be estimated by considering renal function (SUA/sCr). Elevated uric acid levels in normal or mildly impaired renal function are highly suggestive of abnormal uric acid production or tubular reabsorption, which can directly or indirectly contribute to the dangerous vascular effects of hyperuricemia (31). Notably, Insulin Resistance(IR) is associated with both hypertension and hyperuricemia, Previous studies report increased frequency of insulin resistance and hyperinsulinemia in patients with essential hypertension (32), It has also been shown that insulin resistance is directly related to the severity of hypertension (33), It means that hypertension can have the presence of insulin resistance. IR can also lead to hypertension in people with normal blood pressure. Conclusive evidence suggests that insulin induces water and sodium retention and that both endogenous and exogenous hyperinsulinemia are associated with increased blood pressure. Of the pro-hypertensive mechanisms that may be associated with hyperinsulinemia, those related to the renal antidiuretic effect of insulin appear to be the most relevant. By acting on insulin receptors in the renal tubules, insulin can increase renal reabsorption of sodium, triggering sodium retention and renal volume expansion, which in turn raises blood pressure (34). Hepatic IR, on the other hand, leads to an increase in gluconeogenesis and a decrease in glycogen synthesis, triggering fasting hyperglycemia and an increase in blood pressure (35–37). The above suggests a possible bidirectional causal effect between hypertension and IR. Meanwhile, IR can also lead to hyperuricemia by reducing the ability of the kidneys to excrete urate (38), and an MR study has shown that hyperinsulinemia leads to hyperuricemia and vice versa (39). One study observed a statistically significant difference in median uric acid values between the insulin-resistant and non-insulin-resistant groups (p < 0.001). The median uric acid value for those without IR was 4.6, while the median uric acid value for those with IR was 5.5 (38). It is well known that diuretics have long been used in the treatment of hypertension, and many diuretics often inhibit sodium reabsorption by acting on different parts of the renal tubules and exerting the biological effect of reducing capacity and consequently lowering blood pressure. However, long-term use of diuretics may cause the human body to increase uric acid levels by increasing uric acid reabsorption and decreasing uric acid excretion, or by increasing the production of uric acid, thus inducing hyperuricemia, which in turn might potentially lead to the development of gout. IR works in both directions with hypertension, which is positively associated with hyperuricemia, and hyperuricemia can also lead to IR, resulting in renal impairment of the kidneys, which reduces uric acid excretion leading to hyperuricemia and eventually to gout. In summary, we suggest that the mechanism of causal association between elevated blood pressure and positive association with gout risk is closely related to low insulin resistance. However, some studies have also found that when adjusted for some variables, the positive association between hypertension and gout risk was not significant (40). It should be noted that the inconsistent associations observed in these routine clinical studies are at least partially attributed to differences in sample size, reverse causality, and cultural, dietary, socioeconomic status, lifestyle, and genetic differences between populations at sampling sites that cannot fully mitigate confounding effects. Ignoring any of these contexts may misrepresent the clinical association between blood pressure and gout. Our study, on the other hand, was less likely to be confounded by the above-mentioned relevant factors. Notably, our study revealed the overall effect of blood pressure on gout, and through risk factor analysis, we found that both systolic and diastolic BP were associated with higher BMI, which has been demonstrated in several large-scale observational studies (41), and the causal relationship has recently been confirmed by two MR studies (42, 43).

### Diastolic and systolic blood pressure and unspecified gout, idiopathic gout, strictly defined gout

Our findings support that those with hypertension are more likely to develop unspecified gout, idiopathic gout, and strictly defined gout. There is a lack of current population-based epidemiological and clinical trial studies looking at the association between diastolic and systolic blood pressure and these specific subtypes of gout. Therefore, the interpretation of our MR results is less evidence-based and more speculative, and further work and more independent evidence is needed to validate these results above.

### Significance of the current study

Although current work identifies some causal role for blood pressure in gout, unspecified gout, idiopathic gout, and strictly defined gout, MR results should be interpreted with caution. Methodological researchers caution against interpreting MR causal estimates as the expected effect of interventions on risk factors in the clinical setting (44). Although it may not be appropriate to extrapolate MR results to guide clinical interventions, causal inference using MR designs may be useful in screening specific populations for disease susceptibility. In our study, we found that people with hypertension are more likely to develop gout, and as IR may play a key role in advancing this process, we recommend that when a patient has a tendency to have elevated blood pressure, prompt attention should be given to reducing the onset of IR. There are no medications specifically approved for the treatment of insulin resistance, so we focus more on reducing the incidence of IR through dietary changes, increased exercise, reduced smoking and drinking, and other modifiable lifestyle changes. A low-salt, low-fat, high-protein diet is especially helpful for people trying to improve insulin sensitivity. Regular physical activity of approximately 30 minutes on at least 5 days per week leads to muscle cell activation, and increased AMPK activity, which inactivates TCB 1D1 and promotes GLUT4 translocation to the cell membrane, in addition to increased glucose uptake, which increases insulin responsiveness (37). Thus more frequent routing screening of gout and more stricter lifestyle interventions are recommended in those with blood pressure. In addition, the association relationship we identified between blood pressure and multiple gout phenotypes also provides strong support for further conducting randomized controlled trials.

### Strengths and limitations

We used large-scale GWAS data to ensure sufficient sample size and appropriate genetic phenotypes to explore the causal relationship between blood pressure and gout. As a novel medical analysis method similar to RCT, MR has been recognized in recent years as effective in reducing bias and can be used to uncover causal relationships. In conclusion, using large-scale genetic pooled data, our study found evidence of a causal relationship between blood pressure and four gout phenotypes. We believe there is a need to further investigate the underlying mechanisms between blood pressure and gout at the metabolic level.

Some limitations should also be taken into account in our study. Firstly, as this study was conducted among participants of European origin, the results cannot be immediately generalized to other ethnic groups with different lifestyles and cultural backgrounds. Second, because MR analyses infer causal hypotheses by using random assignment of genetic variants, it is difficult to fully distinguish between mediation and pleiotropy using MR methods. A large number of variants in our genome may affect one or more phenotypes. Meanwhile, our chosen statistical method of MR can only provide a reliable estimate of the causal effect of exposure, and cannot prove causality, as only experimentation is the gold standard for causality, and thus more and further experimental evidence is needed to validate our results. Third, our data is from a public database, is a group of data, and is not specific to individual cases, so the results should be interpreted carefully, our results highlight the effects of genetic predisposition rather than disease status in observational studies. Reassuringly, our sensitivity analysis did not detect any existence of pleiotropy biasing our results. Last, additional mediation analyses and observational studies are lacking to further identify the metabolic mechanisms involved in the causal relationship between blood pressure and Gout-related phenotypes.

To date, the development for the prevention of gout remains a challenge for clinicians. Previous studies have mostly concluded that gout increases the risk of hypertension, and the underlying pathogenesis is being extensively investigated (45). However, there is little evidence on the causal role of hypertension in gout development and even less research on the causal relationship of systolic and diastolic blood pressure with gout. Thereby, we used an MR design to investigate the causal relationship between systolic and diastolic blood pressure and gout, drug-induced gout, idiopathic gout, unspecified gout, and strictly defined gout. In genetic predisposition, our results suggest that both systolic and diastolic blood pressure play a key role in the development of gout.

## Conclusion

Our study demonstrates that diastolic and systolic blood pressure exerts a diverse and widespread impact on gout-related phenotypes, including gout, idiopathic gout, nonspecific gout, and strictly defined gout. These findings may have implications for the establishment of feasible screening and prevention strategies for gout disease in patients with high blood pressure. To prevent gout and its comorbidities, high attention should be paid to blood pressure control.

## Data availability statement

The original contributions presented in the study are included in the article/**Supplementary Material**, further inquiries can be directed to the corresponding authors.

## Author contributions

YL: Writing – original draft. YX: Writing – review & editing. JL: Writing – review & editing, Data curation. ZC: Writing – review & editing, Data curation. JZ: Writing – review & editing, Data curation. ZZ: Writing – review & editing, Data curation. RR: Writing – review & editing. YC: Writing – review & editing.
