# Supplementary material for: Diastolic and systolic blood pressure and gout: a Mendelian randomization study
Source: Front Endocrinol (Lausanne). 2024 May 22;15:1367621. doi: 10.3389/fendo.2024.1367621 (PMC11150642; doi:10.3389/fendo.2024.1367621)

Leave-one-out analysis for all significant causal relationships detected in primary analysis. Within Each panel, the black points represent the causal estimate of association between a specific exposure and target mental disorder after discarding each SNP in turn. Red points represent the overall causal estimate using the random-effects inverse variance weighted. Horizontal lines denote 95%confidence intervals.


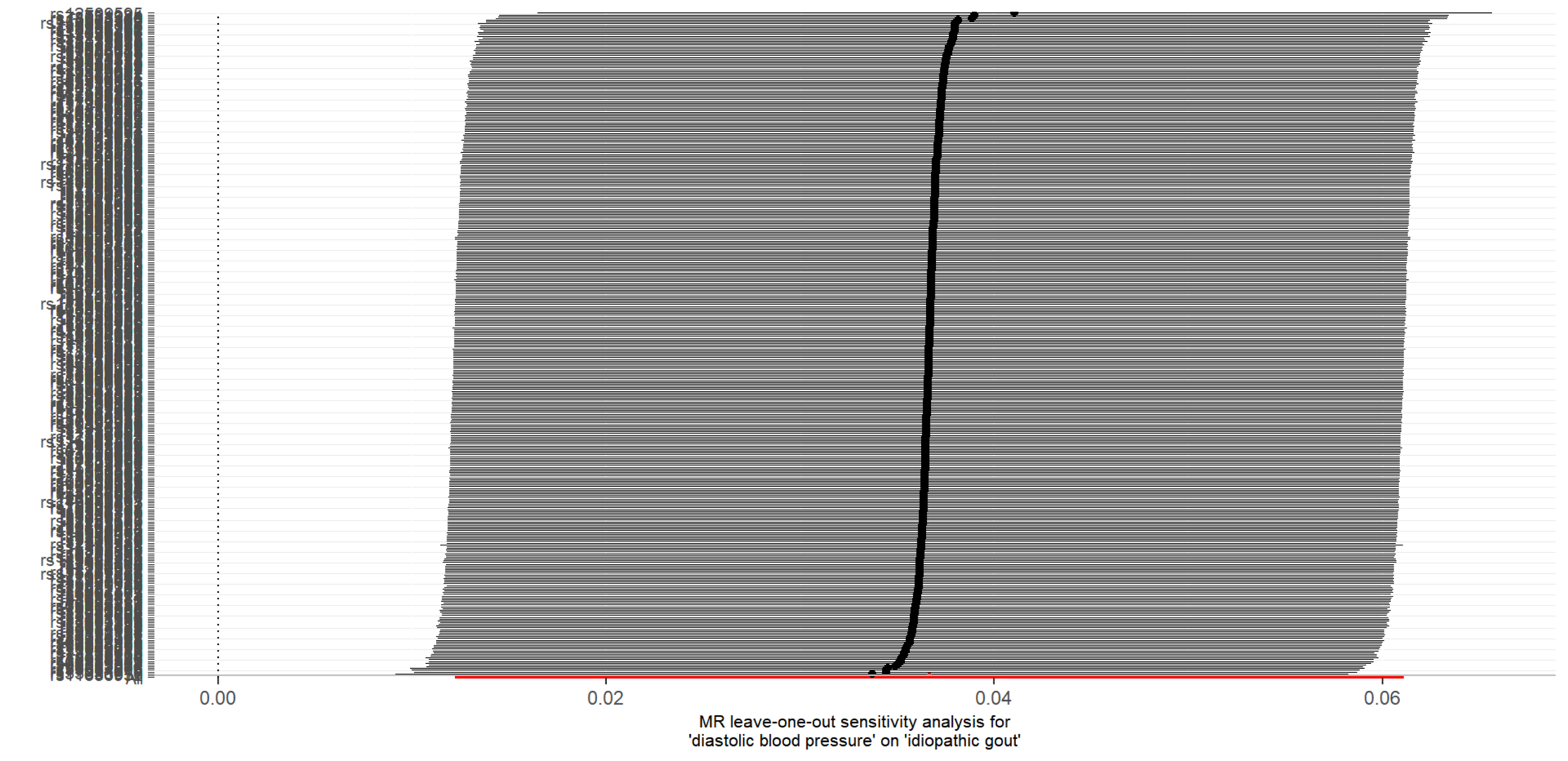

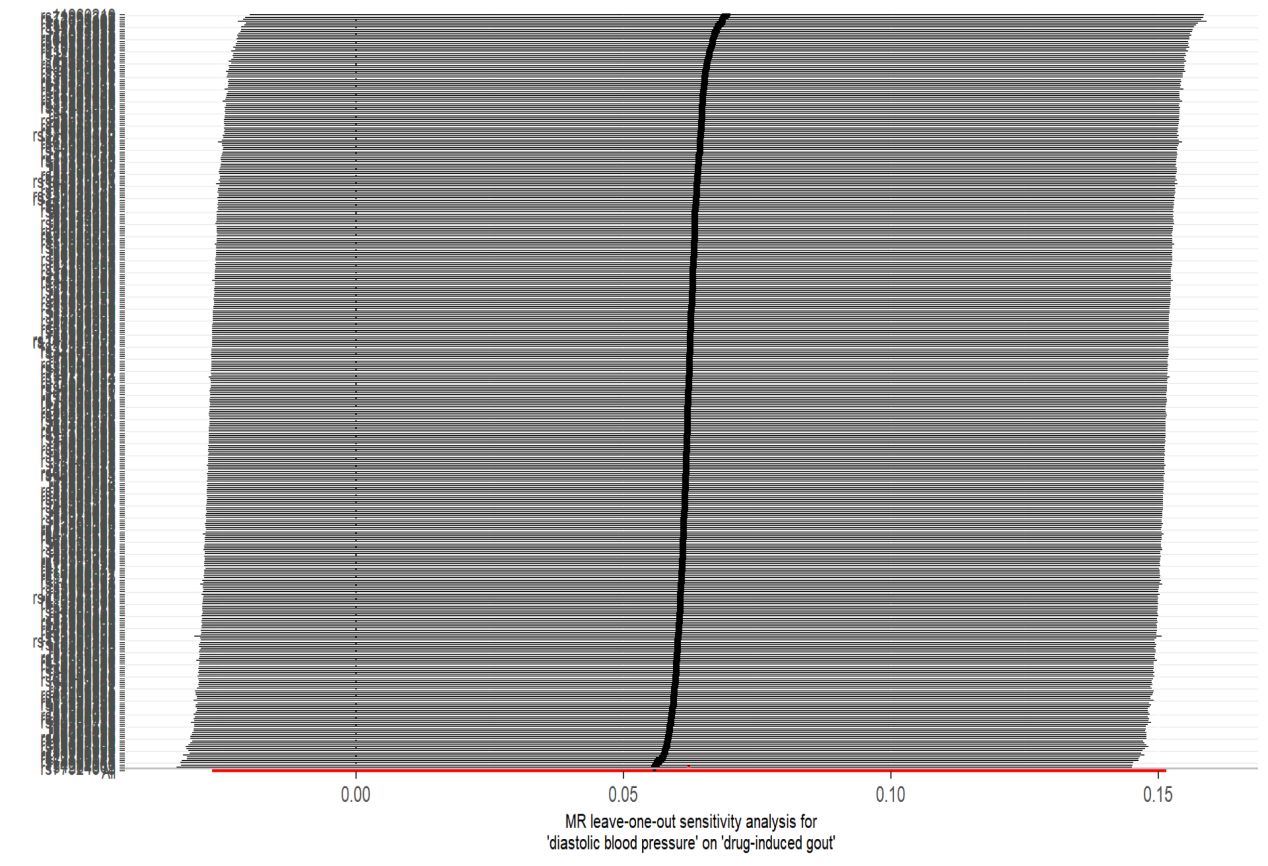

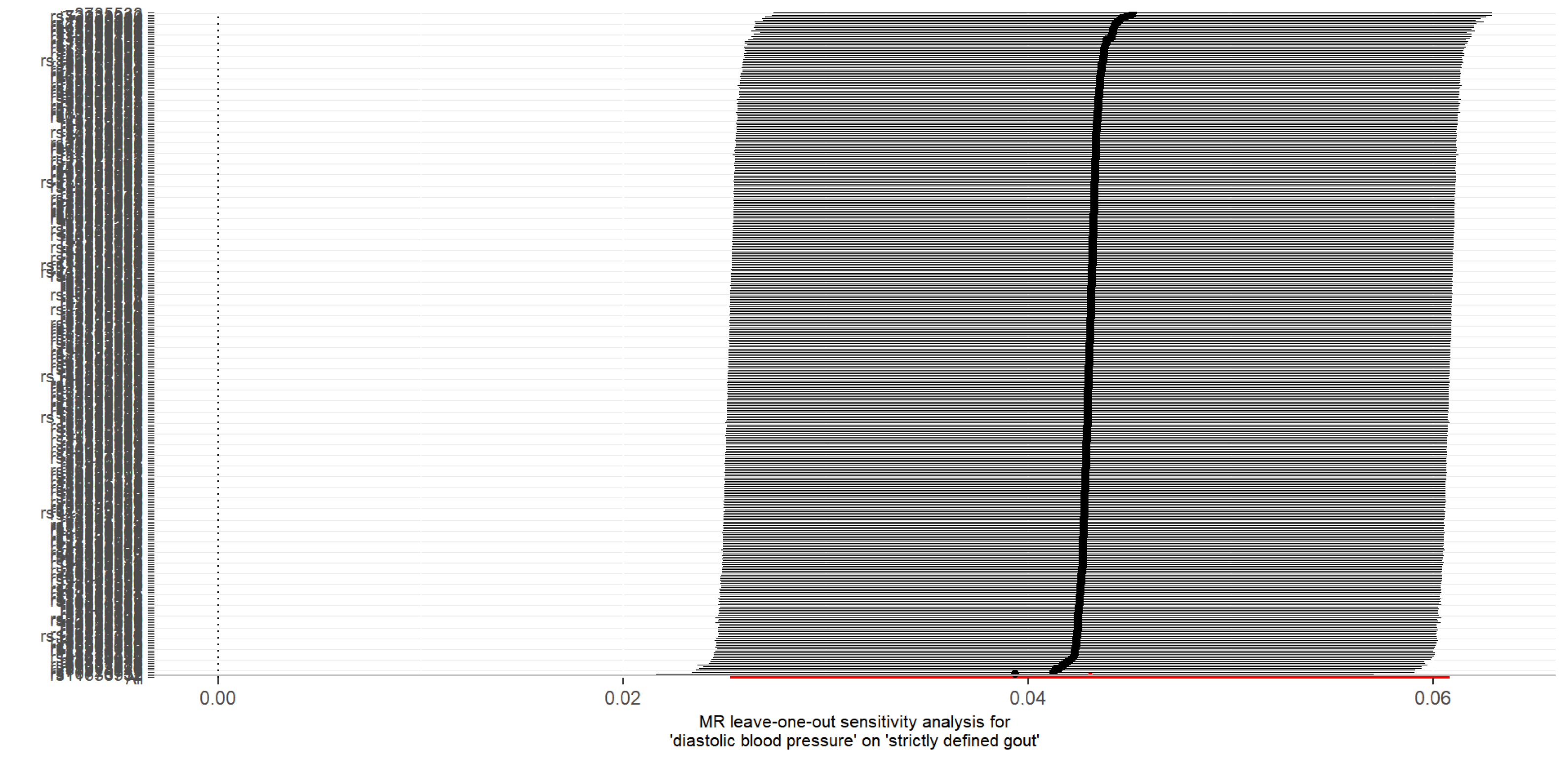

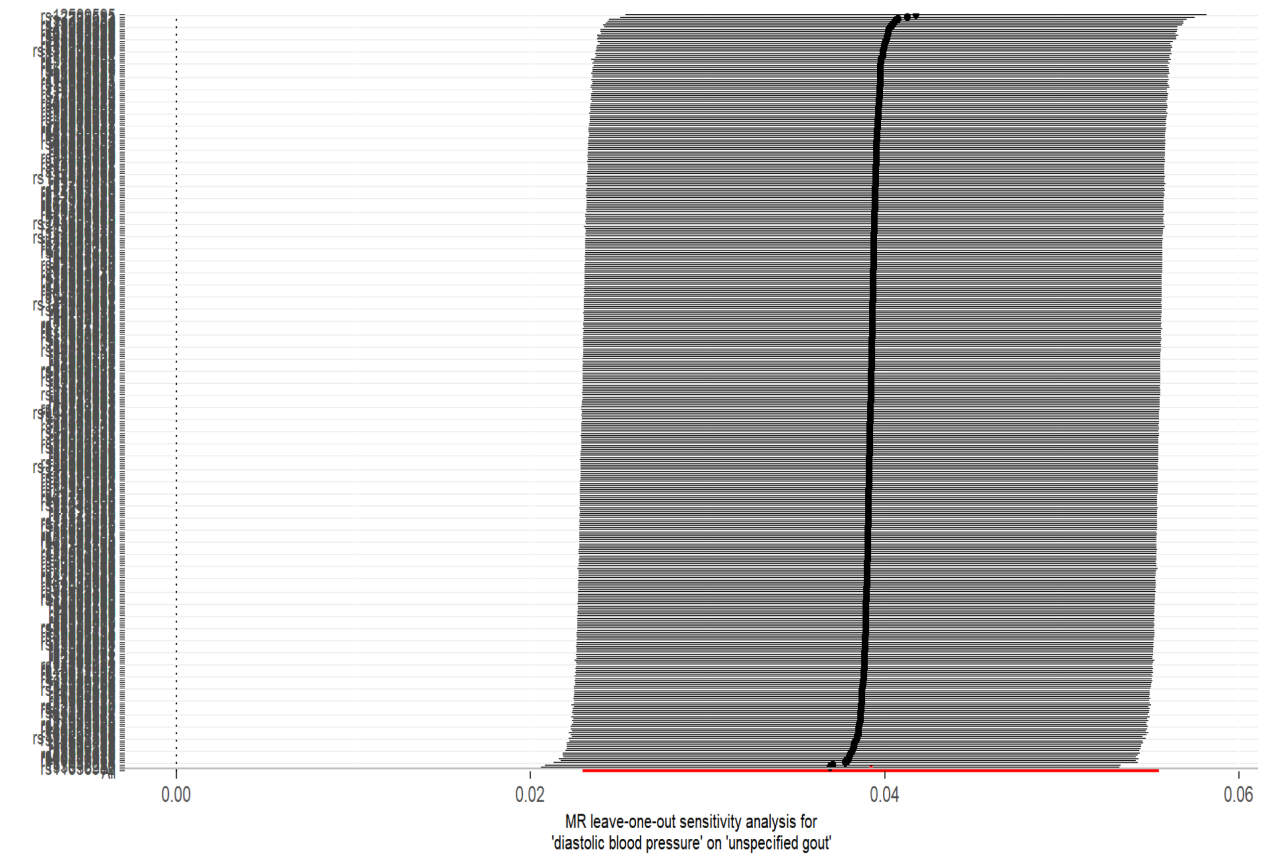

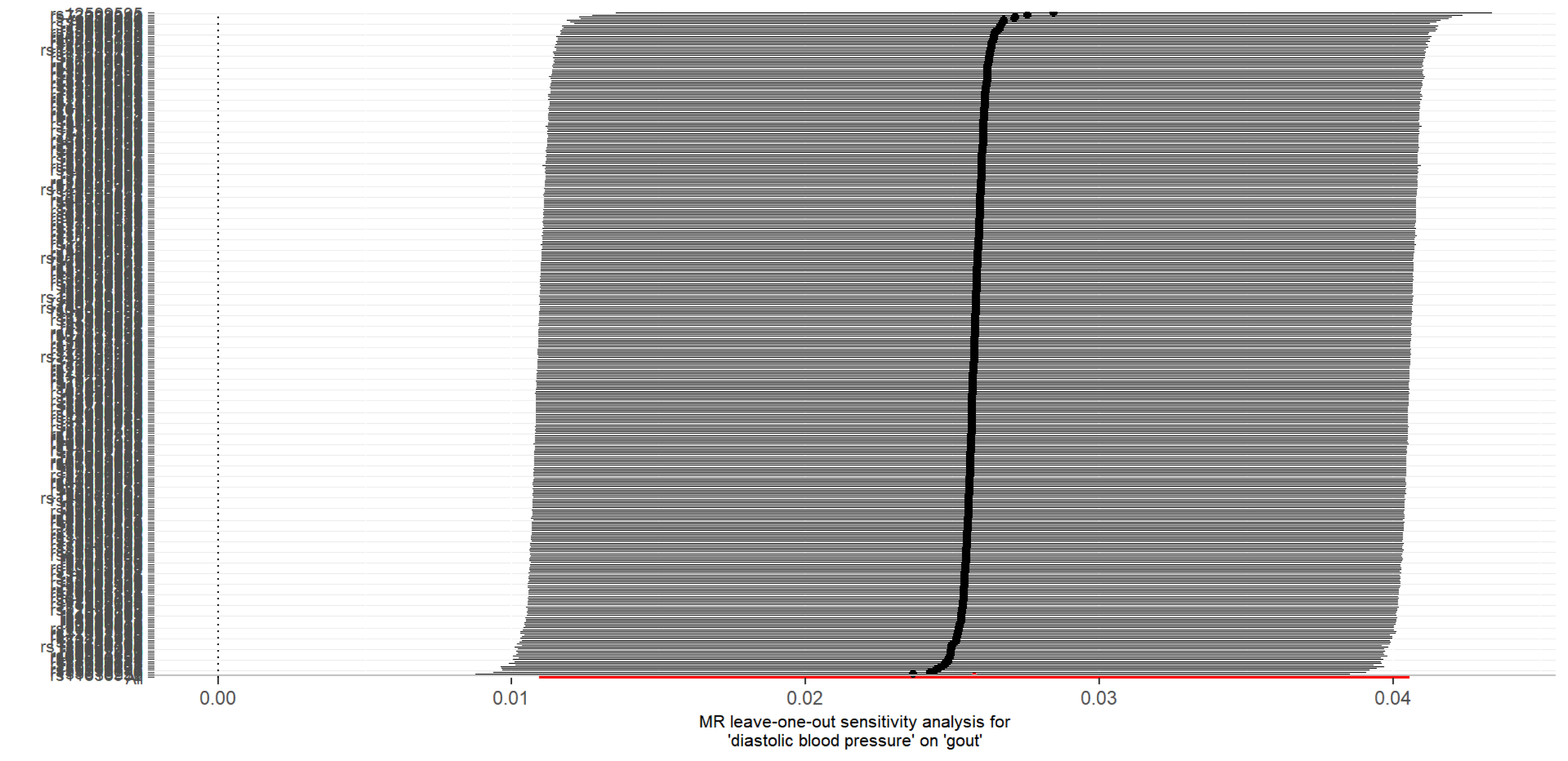

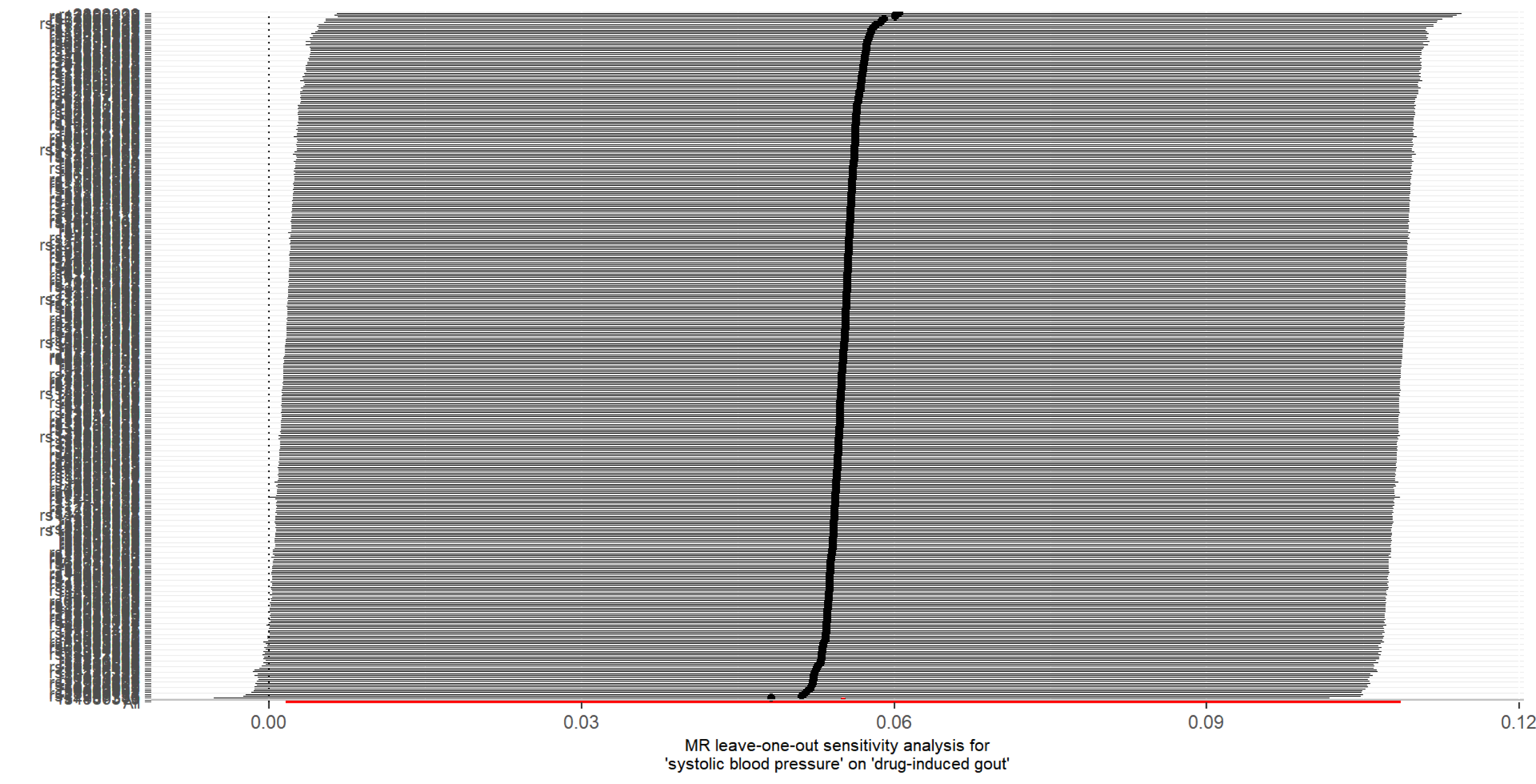

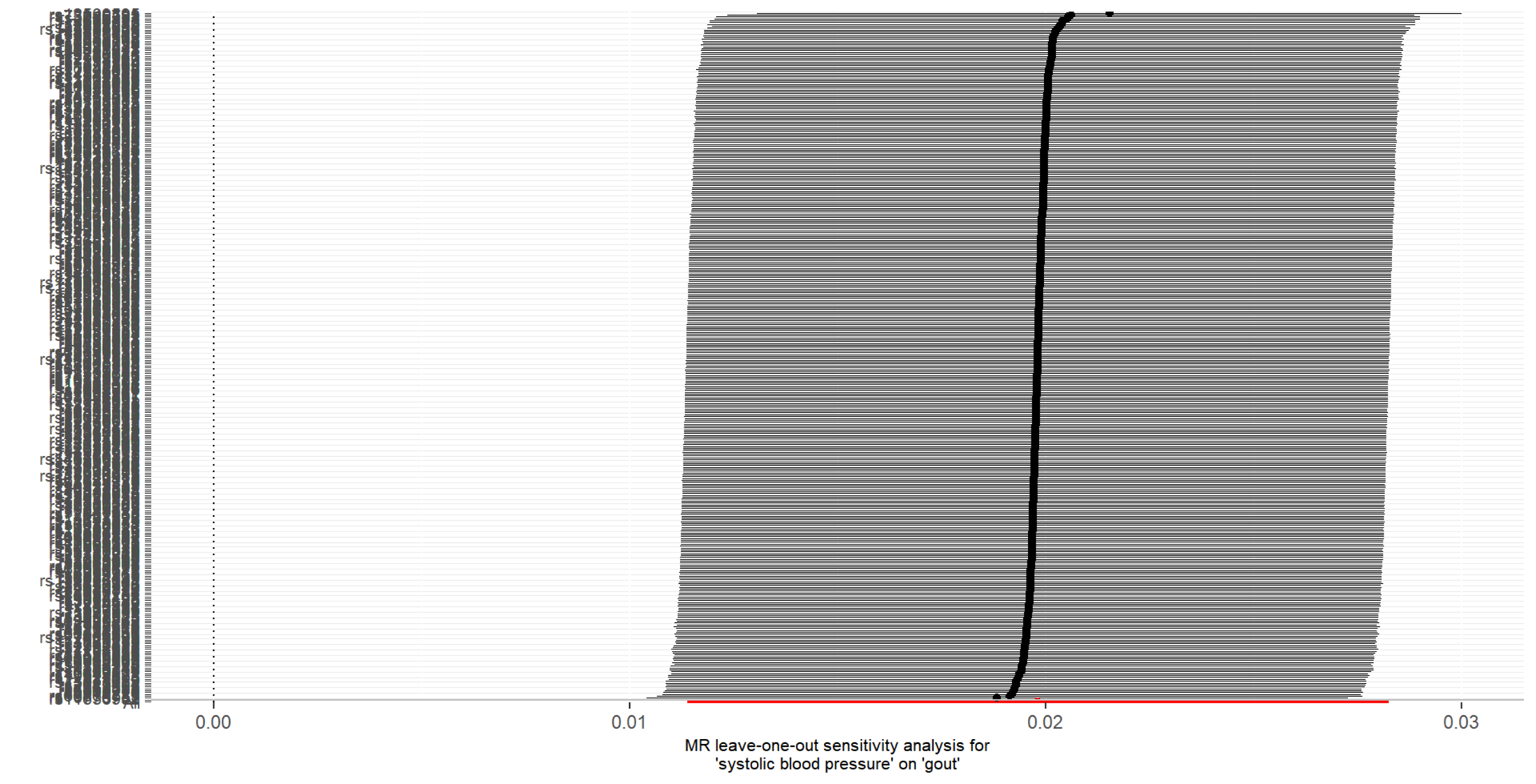

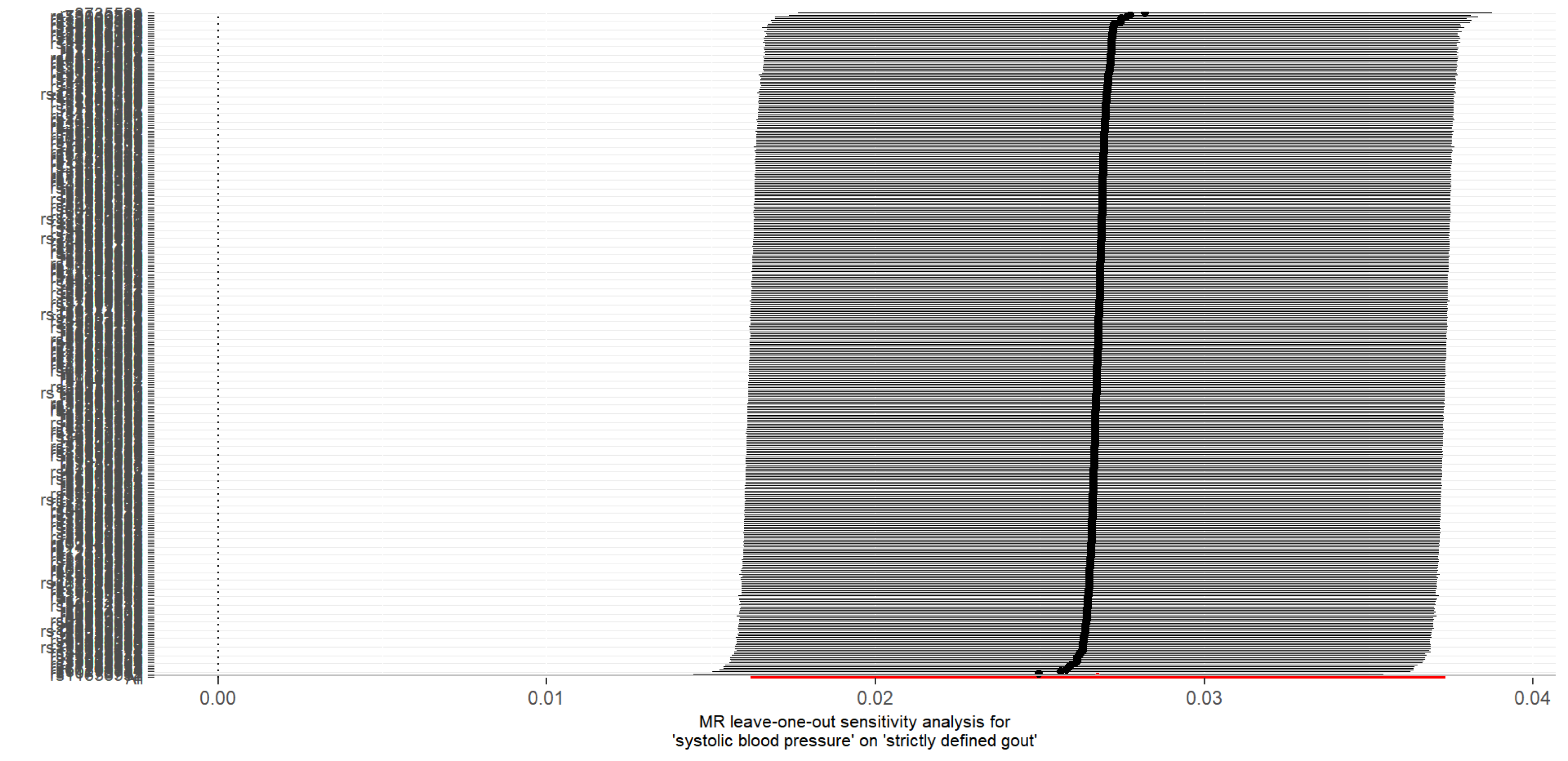

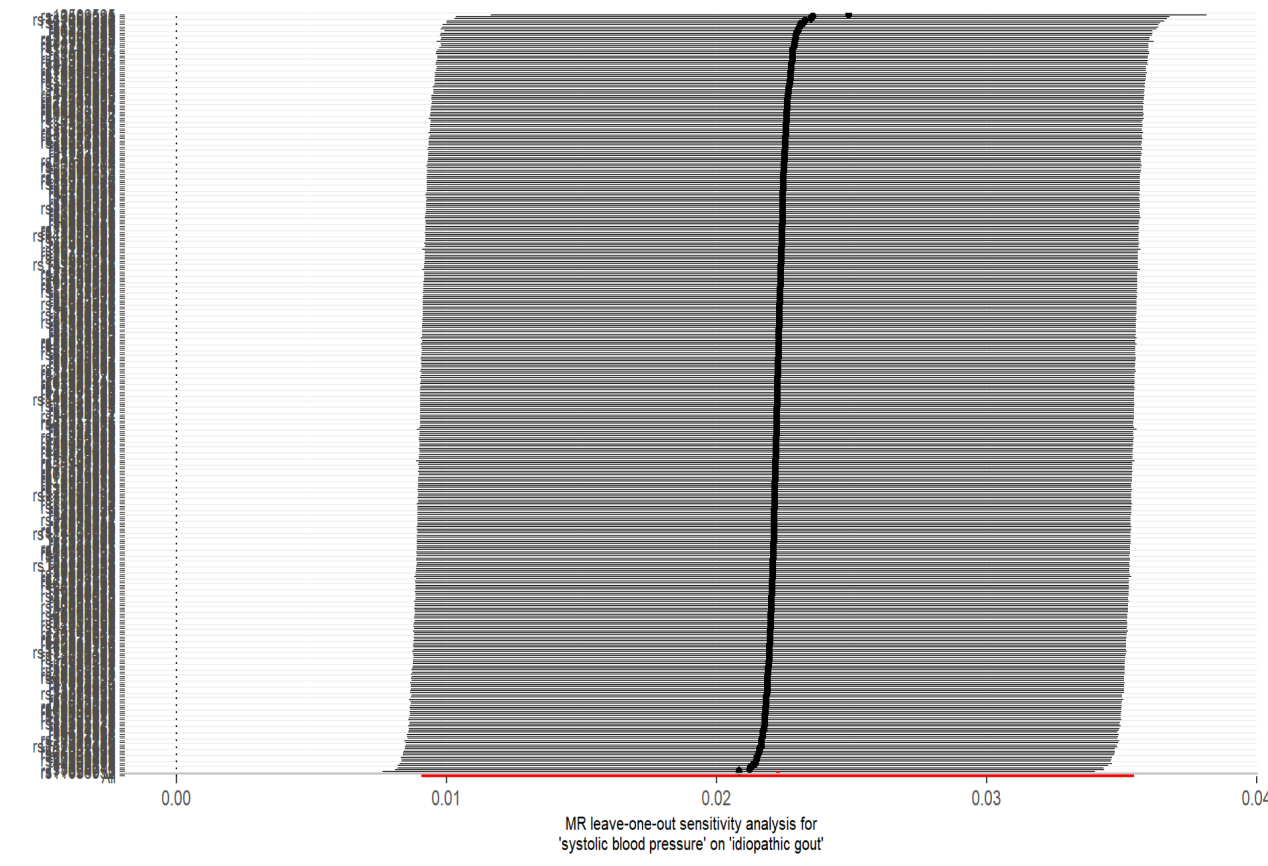

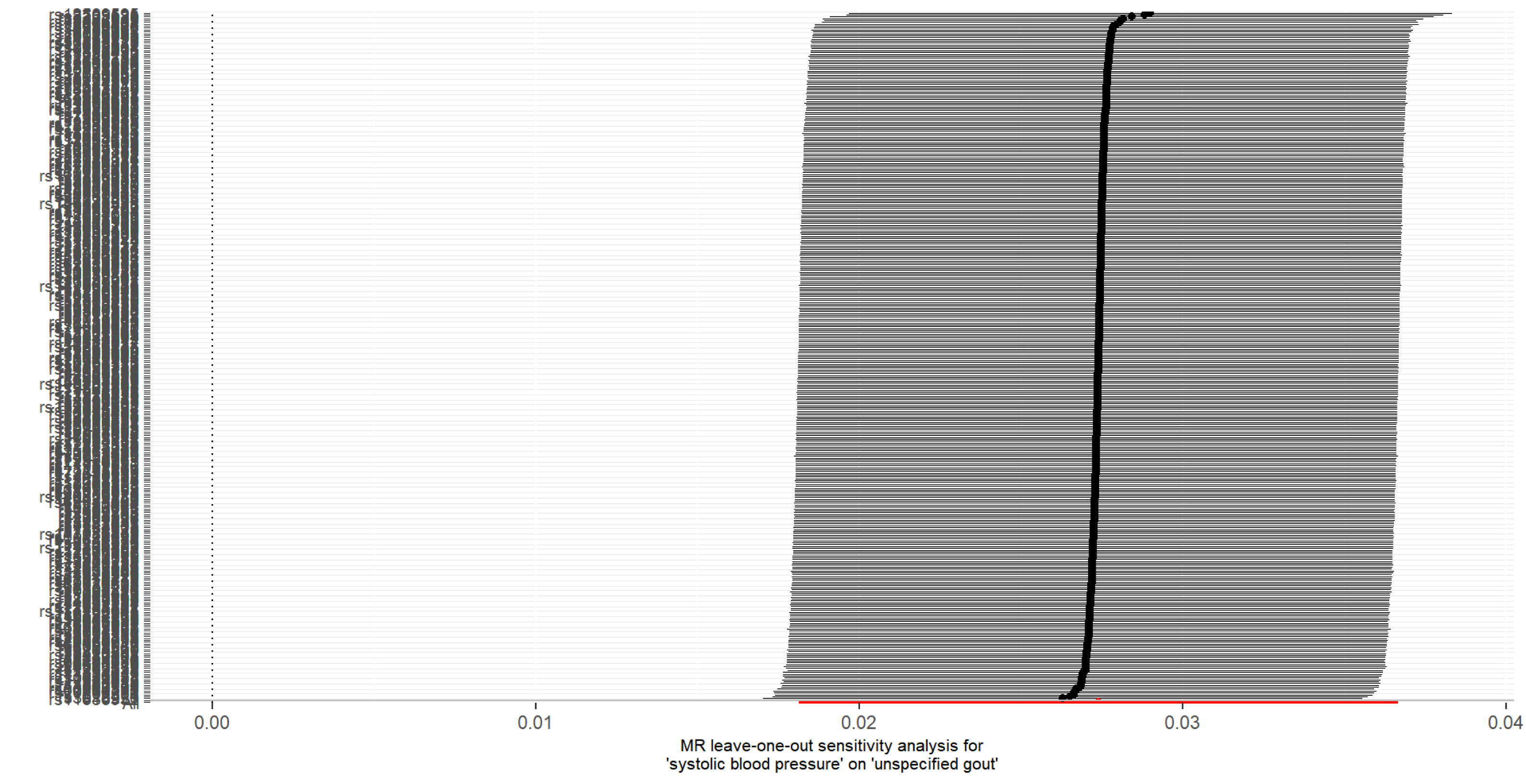

Supplement: Supplementary file 2 [file DataSheet_1.docx]
